# Nano-Power OTA-Based Low-Pass Filter for Ultra-Low-Energy Biomedical Signal Processing

**DOI:** 10.3390/s26092586

**Published:** 2026-04-22

**Authors:** Tomasz Kulej, Montree Kumngern, Fabian Khateb

**Affiliations:** 1 Department of Electrical Engineering, Czestochowa University of Technology, 42-201 Czestochowa, Poland; kulej@el.pcz.czest.pl; 2 Department of Telecommunications Engineering, School of Engineering, King Mongkut’s Institute of Technology Ladkrabang, Bangkok 10520, Thailand; montree.ku@kmitl.ac.th; 3 Department of Microelectronics, Brno University of Technology, Technická 10, 601 90 Brno, Czech Republic; 4 Department of Electrical Engineering, Brno University of Defence, Kounicova 65, 662 10 Brno, Czech Republic

**Keywords:** OTA, ultra-low-power circuits, subthreshold CMOS, low-pass filter, biomedical signal processing, ECG conditioning

## Abstract

This paper presents a nanowatt-scale operational transconductance amplifier (OTA) and an electronically tunable third-order low-pass filter (LPF) designed for energy-constrained biomedical signal conditioning. The circuits are implemented in a 65 nm CMOS process and verified through comprehensive schematic-level simulations. Biased in the deep subthreshold region at 1 nA, the OTA achieves a 50 dB low-frequency gain, a 225 Hz unity-gain bandwidth at 10 pF load capacitance and an input-referred noise floor of 1.55 μV/√Hz, with a total power consumption of only 1.75 nW. The integrated third-order LPF provides a wide tuning range (37–668 Hz) via bias current modulation, exhibiting excellent linearity with a THD of 0.059% and a 65.3 dB dynamic range. Monte Carlo and PVT corner analyses demonstrate the design’s theoretical robustness against process variations and environmental fluctuations. ECG signal simulations validate the circuit’s effectiveness in suppressing high-frequency artifacts while preserving morphological integrity, providing a proof-of-concept for ultra-low-power wearable healthcare architectures.

## 1. Introduction

The proliferation of wearable and implantable health-monitoring devices has intensified the demand for ultra-low-power (ULP) analog front-end (AFE) circuits. These systems, designed for the acquisition of biopotentials such as ballistocardiograms (BCG), electrocardiograms (ECG), and electroencephalograms (EEG), must operate under stringent energy constraints while maintaining high signal fidelity. Specifically, the AFE must amplify and filter low-frequency, microvolt-level signals while minimizing the Power Efficiency Factor (PEF) and Noise Efficiency Factor (NEF) [1,2]. Achieving nanowatt-scale operation without compromising gain, linearity, or noise performance remains a significant challenge in modern CMOS analog design [3].

Operational transconductance amplifiers (OTAs) serve as the fundamental building blocks for continuous-time $G_{m}-C$ filters. In these architectures, the electronically tunable transconductance ($g_{m}$) of the OTA directly dictates the filter’s cutoff frequency and overall transfer function [1,4]. To achieve nanowatt-level dissipation, transistors are typically biased in the deep subthreshold (weak inversion) region, where $g_{m}$ is linearly proportional to the bias current $I_{B}$ divided by the thermal voltage $V_{T}$. This biasing strategy provides the maximum possible $g_{m}/I_{D}$ ratio, which is critical for low-frequency biomedical applications where power budgets are severely limited [1,3].

Considerable research has focused on bulk-driven (BD) OTA architectures to facilitate low-voltage operation. By applying the input signal to the transistor body, BD designs circumvent threshold voltage ($V_{th}$) limitations, enabling near rail-to-rail input common-mode ranges [5,6]. However, the BD approach inherently suffers from reduced effective transconductance ($g_{mb}$ approx. 0.15 × $g_{m}$ for the technology used) and elevated input-referred noise compared to traditional gate-driven (GD) structures [5,6]. While recent BD enhancements have improved linearity [7], the diminished $g_{mb}$ often necessitates higher bias currents to achieve comparable gain, potentially offsetting the power advantages in precision AFEs.

Conversely, gate-driven OTAs leverage the intrinsic $g_{m}$ of the MOSFET, offering superior gain-per-unit-current and lower noise floors. In the context of 65 nm CMOS technology, GD architectures provide a robust platform for capturing weak biopotentials. By utilizing subthreshold techniques alongside linearization methods—such as local feedback or composite transistors—GD OTAs can achieve high linearity and stable transconductance at nanowatt levels [1,3,4]. Furthermore, inverter-based OTAs have emerged as compact, energy-efficient alternatives by utilizing current-reuse techniques; however, they may exhibit higher sensitivity to supply voltage fluctuations and process variations in advanced nodes [8,9].

This paper proposes a high-performance, gate-driven OTA and a tunable third-order Gm-C low-pass filter (LPF) implemented in a 65 nm CMOS process. This technology node was selected to achieve a favorable balance between integration density, power efficiency, and reliability for subthreshold operation. Compared to older 180 nm nodes, the 65 nm process offers a significantly smaller silicon footprint, which is critical for modern portable and implantable biomedical devices. Furthermore, 65 nm provides more stable subthreshold characteristics and lower gate leakage compared to deeper sub-micron nodes (e.g., below 40 nm), making it an ideal platform for nanowatt analog signal processing. The design is optimized to bridge the gap between extremely low power consumption and high dynamic range. Through comprehensive schematic-level simulations, including Monte Carlo and PVT corner analyses, this work demonstrates that a meticulously biased GD OTA can achieve a 50 dB gain and a sub-kilohertz tunable bandwidth with a total power footprint of only 1.75 nW. The resulting third-order filter provides the steep roll-off and noise suppression required for high-fidelity BCG/ECG/EEG acquisition, offering a theoretically robust proof-of-concept for next-generation biomedical sensing.

## 2. Circuit Description

### 2.1. Proposed OTA Circuit

**Figure 1:**
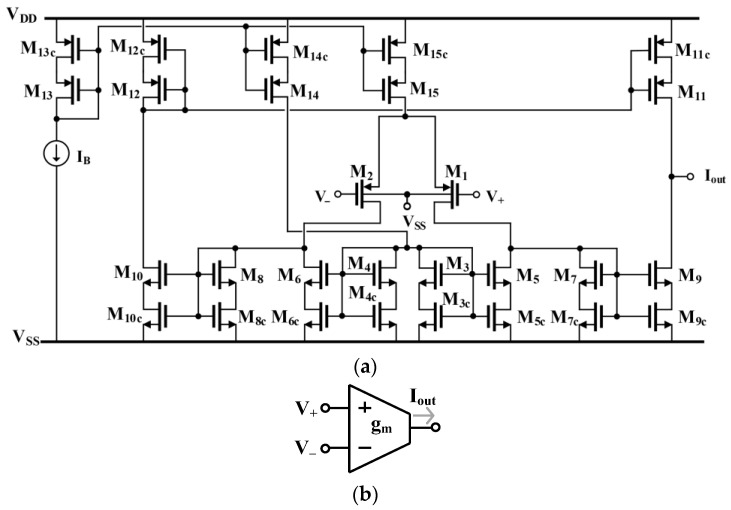
Circuit schematic of the OTA (**a**) and electrical symbol (**b**).

The schematic diagram of the operational transconductance amplifier (OTA) is shown in Figure 1a, and its electrical symbol is shown in Figure 1b. The input stage employs a classical MOS differential pair (M_1_ and M_2_) conventionally driven from the gate terminals. In order to reduce the threshold voltages of the transistors, the bulk terminals of these devices are connected to the lowest potential in the circuit ($V_{SS}$). Considering the expected supply voltage of ±0.25 V, the V_BS_ voltage of the input transistors in this configuration will always remain below 0.5 V, thus preventing the occurrence of the latch-up effect.

Taking into account the assumed very small bias currents ($I_{B}=$1 nA) and the reduction in the threshold voltage due to the body effect, the $V_{SG}$ voltages of transistors M1 and M2 are only on the order of 10–20 millivolts at the operating point, while the nominal threshold voltages of p-channel transistors in the applied 65 nm technology are −0.36 V. Such a small value of $V_{SG}$ at the operating point allows a relatively large range of input common-mode voltages, approximately ±100 mV, despite gate-driven operation and the relatively high ratio of threshold voltage to supply voltage.

The differential transfer characteristic of the input pair operating in the weak inversion region can be described by [10]

(1) $$I_{D2}-I_{D1}=I_{B}\tanh\left(\frac{V_{+}-V_{-}}{2n_{p}U_{T}}\right)$$

where $n_{p}$ is the emission coefficient for the p-channel transistor, $U_{T}$ is the thermal voltage, and $I_{B}$ is the bias current (assuming that the transistors forming the bias current source M_15,15c_ are identical to transistors M_13,13c_).

The small-signal transconductance of the differential pair can be expressed as

(2) $$g_{m}=\frac{I_{B}}{2n_{p}U_{T}}$$

In general, the entire amplifier structure is based on the architecture known as a current mirror OTA. The current mirrors forming the structure (M_3_–M_11_) employ self-cascode (SC) composite transistors. This solution increases the output resistance and consequently the voltage gain of the entire OTA, while maintaining relatively small compliance voltages for each current mirror.

The drain voltages of transistors M_3_–M_8_ are set to approximately 150 mV at the operating point, corresponding to about 75 mV $V_{DS}$ for each (bottom and stacked) transistor in the SC pair. This ensures operation at the boundary of the saturation region and provides output resistances comparable to those of classical cascode connections.

The output resistance of the M_i_–M_ic_ connection can be expressed as

(3) $$r_{out,i}≅r_{ds,i}\left(1+g_{m,i}r_{ds,ic}\right)$$

To further increase the voltage gain of the circuit, the input transistors of the current mirrors in the load of the input stage are shunted by constant current sinks [11]. In the circuit shown in Figure 1, the pairs M_7,7c_ and M_8,8c_ are shunted by current sinks formed by composite transistors M_5,5c_ and M_6,6c_, respectively.

It should be emphasized that the currents of the shunting transistors must always be smaller than the currents of the input transistors of the current sinks being shunted. This condition must be satisfied for process, supply voltage, and temperature variations within the assumed range, as well as for possible transistor mismatches.

In the design, it was assumed that the ratio of the drain currents of transistors M_5,6_ to the drain currents of transistors M_7,8_ is equal to m, where m< 1. This factor is ensured by an appropriate biasing scheme. The current source M_14,14c_ ($I_{B}$) biases transistors M_3,3c_ and M_4,4c_, which together with M_5,5c_ and M_6,6c_, form current mirrors. It was assumed that M_3,3c_ and M_4,4c_ are identical to transistors M_7,7c_–M_10,10c_.

The desired value of coefficient m is obtained by selecting an appropriate ratio of the channel widths of transistors M_5,5c_ and M_3,3c_ (and symmetrically M_6,6c_ and M_4,4c_) equal to m. Furthermore, assuming that pair M_7,7c_ is identical to M_9,9c_, M_8,8c_ is identical to M_10,10c_, and pair M_11,11c_ is identical to M_12,12c_, and neglecting second-order effects, the small-signal transconductance of the OTA can be expressed as

(4) $$g_{m,OTA}=\frac{I_{B}}{2n_{p}U_{T}}$$

Thus, it is equal to the transconductance of the MOS differential input pair. The absence of transconductance change is advantageous in circuits intended for low-frequency applications requiring small values of this parameter.

However, the applied technique affects the output resistance of the OTA, which can be expressed as

(5) $$r_{out,OTA}≅r_{ds9}\left(1+g_{m9}r_{ds9c}\right)||r_{ds11}\left(1+g_{m11}r_{ds11c}\right)$$

Since, in the considered configuration, transistors M_9,9c_–M_12,12c_ are biased with a smaller current ${(1-m)I}_{B}/2$ than the transistors of the input differential pair ($I_{B}/2$), the output resistance of the circuit increases by a factor of 1/(1−m), which leads to an increase in the DC voltage gain

(6) $$A_{vo,OTA}=g_{m,OTA}r_{out,OTA}=\frac{A_{vo,class}}{\left(1-m\right)}$$

where $A_{vo,class}$ is the DC gain of the classical configuration without additional current sources, i.e., without elements M_3,3c_–M_7,7c_ and M_14,14c_.

As follows from (6), the OTA gain increases as m approaches 1 and theoretically becomes infinite at m=1 (neglecting second-order effects). In practice, however, the values of m are limited by second-order effects such as transistor output conductances. The difference 1−m should not be too small; otherwise, it may become negative under transistor mismatch conditions.

This difference also affects the voltage gain of the differential amplifier, since as m approaches 1, the load resistance increases, thereby increasing the voltage gain from the differential input to the differential output between the drains of M_1_ and M_2_. While this effect is not problematic in operational amplifiers, in transconductance amplifiers—where the differential input voltage may be relatively large—excessive gain in the first stage may reduce the linear range by forcing the differential pair transistors or the active load transistors into the triode region for larger input differential voltages.

The voltage gain of the differential amplifier in Figure 1 (the first stage of the OTA) operating in weak inversion is

(7) $$A_{v1st}=\frac{\left(n_{p}/n_{n}\right)}{\left(1-m\right)}$$

where $n_{p}$ and $n_{n}$ are the emission coefficients of the p- and n-channel transistors, respectively. Note that this gain is independent of the bias current $I_{B}$.

For a classical differential pair operating in weak inversion, the allowable input voltage range is strongly limited to about 20 mV for THD = 1%. Therefore, for a given supply voltage, the voltage gain (7) may reach approximately 4–6 *V*/*V* (m= 0.75–0.83), increasing the overall OTA gain by 12–16 dB. This is a significant improvement in deeply scaled 65 nm technology, where intrinsic transistor voltage gains are relatively small.

Another effect limiting the value of m is a parasitic pole associated with the increased output resistance of the first stage. Its value can be approximated by

(8) $$\omega_{p}≅\frac{g_{m7c,8c}}{C_{S}}=\frac{I_{B}}{2n_{n}U_{T}C_{S}}\left(1-m\right)$$

where $C_{S}$ is the sum of parasitic capacitances associated with the drains of transistors M7 and M8 (the differential output of the first stage). Increasing m therefore reduces the frequency of this pole, which occurs at a significantly lower frequency than other parasitic poles associated with the OTA current mirrors.

All parasitic poles of the OTA should occur at frequencies much higher than the cutoff frequency of the integrator implemented using this amplifier and loaded with the assumed capacitance C. This minimizes the phase error of the integrator over a wide range around its cutoff frequency. The ratio of the parasitic pole frequency to the integrator cutoff frequency can be expressed as

(9) $$\frac{\omega_{p}}{\omega_{o}}=\left(1-m\right)\frac{n_{p}}{n_{n}}\cdot \frac{C}{C_{S}}$$

Thus, decreasing the difference 1−m worsens the ratio $\omega_{p}/\omega_{o}$ for a given $C/C_{S}$, increasing the integrator phase error. This effect limits the maximum value of m.

### 2.2. Noise Analysis

Assuming the following MOS transistor noise models for thermal noise (10) and flicker noise (11):

(10) $$\bar{I_{nt}^{2}}=2qI_{D}$$

(11) $$\bar{I_{n1/f}^{2}}=\frac{g_{m}^{2}K_{p,n}}{fC_{OX}\left(WL\right)}$$

where $I_{D}$ is the drain current, q is the elementary charge, $C_{OX}$ is the gate oxide capacitance per unit area, $K_{p,n}$ is the flicker noise constant for p- and n-channel devices, and W and L are the channel width and length, respectively.

Neglecting second-order effects, the equivalent input-referred thermal noise voltage of the OTA is

(12) $$\bar{V_{in,t}^{2}}=\frac{4q\left(2-m\right){\left(2n_{p}U_{T}\right)}^{2}}{I_{B}}$$

while the equivalent flicker noise voltage is

(13) $$\bar{V_{in,1/f}^{2}}=\frac{2}{fC_{OX}}\left[K_{p}\left(\frac{1}{{\left(WL\right)}_{1,2}}+\frac{{\left(1-m\right)}^{2}}{{\left(WL\right)}_{11c,12c}}\right)\;\;+K_{n}{\left(\frac{n_{p}}{n_{n}}\right)}^{2}\left(\frac{{\left(1-m\right)}^{2}}{{\left(WL\right)}_{7c,8c}}+\frac{{\left(1-m\right)}^{2}}{{\left(WL\right)}_{9c,10c}}+\frac{m^{2}}{{\left(WL\right)}_{5c,6c}}\right)\right]$$

As follows from (12) and (13), the input noise depends on coefficient m. For m=0, it equals the noise of a classical OTA (i.e., after removing elements M_3,3c_–M_6,6c_ and M_14,14c_). As m increases, the equivalent input noise decreases because the voltage gain of the first stage increases. Consequently, the noise contribution of transistors M_9,9c_–M_12,12c_ becomes negligible for m close to 1. The proposed structure is therefore noise-efficient.

The noise reduction in the proposed OTA was exactly optimized by addressing both flicker and thermal components based on the models in (12) and (13). To suppress flicker noise, which is dominant at the low frequencies typical of biomedical signals, the gate area (W1,2 × L1,2) of the input transistors was numerically optimized and set to 25 µm^2^ (50 µm/0.5 µm). This ensures that the flicker noise corner frequency is minimized, keeping the noise floor stable within the signal bandwidth. Simultaneously, the thermal noise was optimized by biasing the OTA in the deep subthreshold region to maximize the transconductance efficiency ($g_{m}/I_{D}$). This design choice provides the highest possible transconductance for a given current, effectively minimizing the thermal noise contribution while maintaining a nano-power budget of 1.75 nW.

The total supply current of the amplifier, including the bias circuit ($I_{B}$, M_13,13c_), is

(14) $$I_{DD}=3I_{B}+\left(1-m\right)\frac{I_{B}}{2}$$

Thus, it is only slightly higher than the supply current of the classical OTA without elements M_3,3c_–M_6,6c_ and M_14,14c_, which equals ${3I}_{B}$. Note that for m=1, both currents are identical.

Due to the intended ultra-low-power applications, the circuit was biased with a very small current $I_{B}=\;$1 nA in the nominal case, which, for a supply voltage of ±0.25 V, results in a total power dissipation of approximately 1.7 nW.

### 2.3. Proposed Third-Order Low-Pass Filter

**Figure 2:**
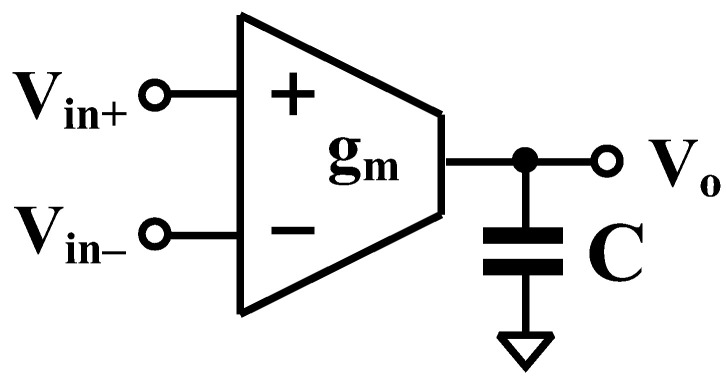
General configuration to realize lossless or lossy integrators.

The proposed OTA is utilized to realize a third-order Butterworth low-pass filter, which can be synthesized from an *n*th-order all-pole low-pass transfer function. The implementation of an nth-order low-pass filter requires subcircuits that realize either lossless or lossy integrators. Figure 2 illustrates the general configuration for the realization of lossless or lossy integrators using OTAs. By straightforward analysis, the output voltage is given by

(15) $$v_{o}=\frac{g_{m}}{sC}\left(v_{in+}-v_{in-}\right)$$

Depending on the configuration of the input terminals, the circuit is capable of realizing both lossless and lossy integrators.

In general, the voltage transfer function of an *n*th-order all-pole low-pass filter can be expressed as

(16) $$H(s)=\frac{V_{out}\left(s\right)}{V_{in}\left(s\right)}=\frac{1}{b_{n}s^{n}+b_{n-1}s^{n-1}+b_{n-2}s^{n-2}+\cdots +b_{2}s^{2}+b_{1}s+1}$$

where $b_{n}$, $b_{n-1},\ldots ,b_{2}$, $b_{1}$ are positive real constants.

**Figure 3:**
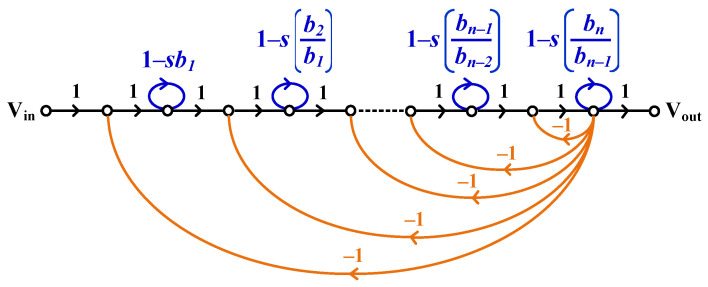
Signal flow graph representing an nth-order all-pole transfer function.

The signal flow graph (SFG) corresponding to the *n*th-order low-pass transfer function in (16) is illustrated in Figure 3. It is evident that the realization employs *n* lossless integrators connected in cascade, together with negative feedback.

Previously reported nth-order low-pass filters can be found in the literature; for example, see [12,13,14,15,16,17,18,19,20,21,22,23]. These filters have been implemented using various active devices, including OTAs [12,13], current conveyors [14,15,16], current feedback operational amplifiers (CFOAs) [16], current follower transconductance amplifiers (CFTAs) [18], digitally programmable current differencing transconductance amplifiers (DPCDTAs) [18], cascaded current differencing units (CCDUs) [20], current differencing cascaded transconductance amplifiers (CDCTAs) [21], voltage differencing gain amplifiers (VDGAs) [22], and current differencing current conveyors (CDCCs) [23]. However, these active devices typically require relatively high supply voltages and exhibit comparatively high power consumption, making them unsuitable for ultra-low-energy biomedical signal processing applications.

**Figure 4:**
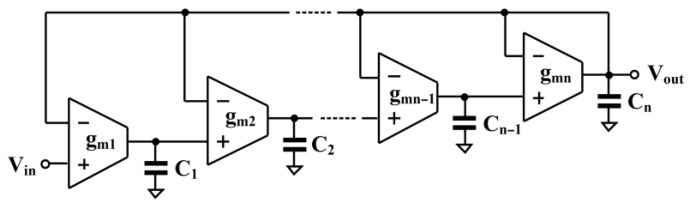
*n*th-order all-pole low-pass transfer function realization using OTAs.

Based on the circuit in Figure 2, the general nth-order all-pole low-pass filter is realized in Figure 4, requiring n OTAs and n capacitors. To suit ultra-low-energy biomedical signal processing applications, a third-order low-pass filter is designed. The transfer function can be expressed as

(17) $$\frac{V_{out}\left(s\right)}{V_{in}\left(s\right)}=\frac{1}{b_{3}s^{3}+b_{2}s^{2}+b_{1}s+1}$$

**Figure 5:**
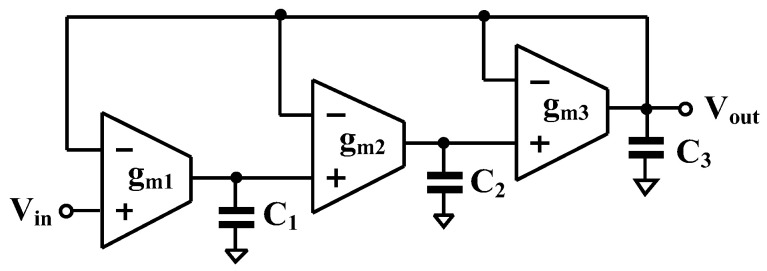
Proposed third-order low-pass filter using OTAs.

For third-order Butterworth polynomial (normalized, $\omega_{c}=1$), the coefficient values are: $b_{3}=1$, $b_{2}=2$, $b_{1}=2$. Using Figure 5, third-order Butterworth low-pass filter is designed as shown in Figure 5. The transfer function can be expressed as

(18) $$\frac{V_{out}\left(s\right)}{V_{in}\left(s\right)}=\frac{\left(\frac{g_{m1}g_{m2}g_{m3}}{C_{1}C_{2}C_{3}}\right)}{s^{3}+\left(\frac{g_{m3}}{C_{3}}\right)s^{2}+\left(\frac{g_{m2}g_{m3}}{C_{2}C_{3}}\right)s+\left(\frac{g_{m1}g_{m2}g_{m3}}{C_{1}C_{2}C_{3}}\right)}$$

By comparing with the normalized values, the following relationships are obtained: ${C_{1}/g_{m1}=b}_{1}=2$, $C_{2}/g_{m2}=b_{2}/b_{1}=1$, and $C_{3}/g_{m3}=b_{3}/b_{2}=0$.5.

By letting the normalized transconductances be $g_{m1}=g_{m2}=g_{m3}=1\;S$, the corresponding normalized capacitances are obtained as $C_{1}=2$ F, $C_{2}=1$ F, and $C_{3}=0.5$ F.

For a designed cutoff frequency of 150 Hz, the transconductance values are chosen as $g_{m1}=g_{m2}=g_{m3}=14.47\;\mathrm{nS}$. Accordingly, the capacitance values are calculated as $C_{1}=30.7$ pF, $C_{2}=15.35$ pF, and $C_{3}=7.38$ pF.

## 3. Results

The proposed OTA circuit was designed and verified through schematic-level simulations in the Cadence Virtuoso environment, targeting the 65 nm TSMC CMOS process. The OTA employs a subthreshold bias current of $I_{B}=1$ nA, resulting in an ultra-low power consumption of 1.75 nW. Regarding the physical implementation, while the current results are based on schematic-level simulations, the impact of layout parasitics is expected to be minimal. Given that the circuit is designed for biomedical applications operating in the sub-kHz frequency range, the parasitic poles and zeros introduced by the layout are located several orders of magnitude above the bandwidth of interest. Furthermore, in this ultra-low-power regime with nanoampere biasing, the performance is primarily governed by transistor sizing and matching rather than parasitic RC effects. This is consistent with previous experimental characterizations of similar bulk-driven, ultra-low-voltage architectures.

The transistor sizing in the implementation is as follows: the input pair transistors M_1_ and M_2_ have a width-to-length ratio W/L (µm/µm) of 50/0.5; M_3_–M_10_ and M_11_-M_13_ are sized at 100/0.2; M_3c_ and M_4c_ have 9/4; M_5c_ and M_10c_ are 4/4 and M_11c_–M_13c_ are 20/4. This sizing ensures proper operation in the subthreshold region while minimizing power consumption, enabling the OTA to meet the requirements of ultra-low-power biomedical applications. The bulk terminals of the input transistors M_1_–M_2_ are connected to VSS to reduce the threshold voltage. Simulation results show that the bulk current is only 13.4 pA, confirming that the parasitic p-n junctions are not forward-biased at the ±0.25 V supply level.

**Figure 6:**
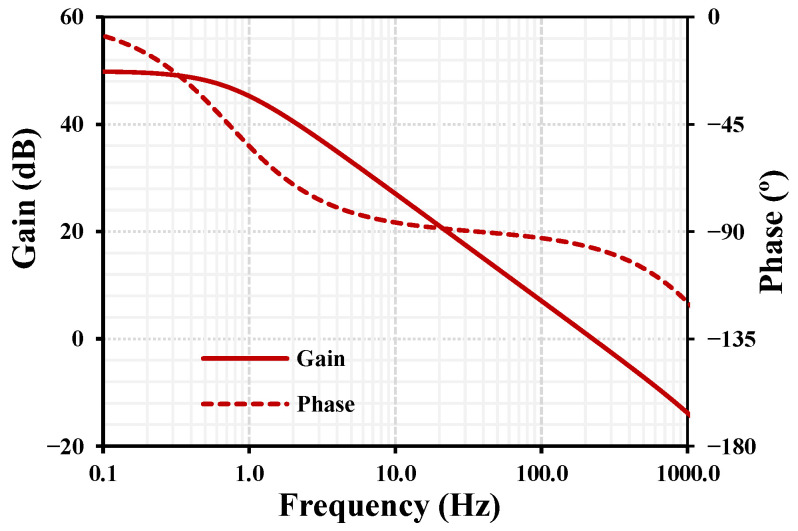
Frequency response of the proposed OTA showing gain and phase characteristics for $C_{L}=10\;\mathrm{pF}$.

The frequency characteristics of the OTA gain and phase with a bias current $I_{B}$ = 1 nA and a load capacitance $C_{L}=10\;\mathrm{pF}$ are shown in Figure 6. The simulated low-frequency gain is approximately 50 dB, and the unity-gain bandwidth (UGB) is 225 Hz. This bandwidth is appropriate for low-frequency biomedical signals, such as ECG and EEG, and results from the extremely small transconductance employed to achieve nano-power operation, which represents a typical trade-off in ultra-low-power biomedical front-end circuits. At the unity-gain frequency, the phase shift is −96.8°, corresponding to a phase error of 6.8° relative to the ideal −90° of a single-pole response. This value indicates good phase behavior and stable operation for nano-power OTA designs targeting low-frequency biomedical signal processing.

**Figure 7:**
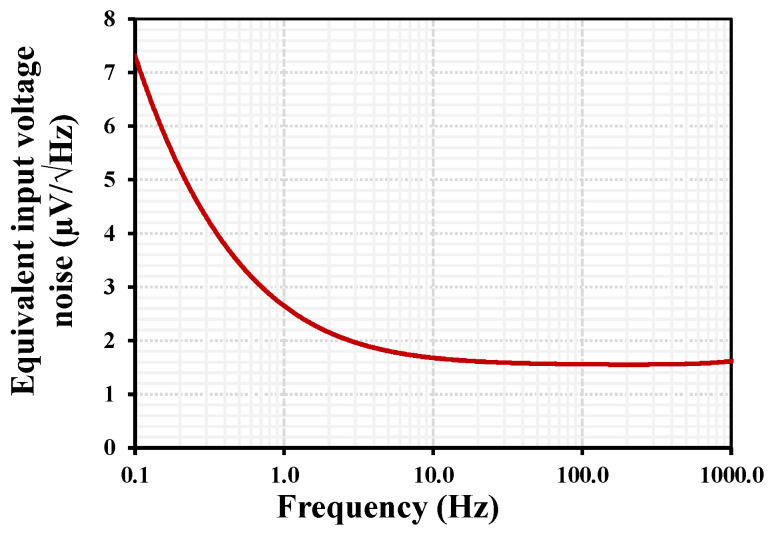
Simulated equivalent input-referred noise voltage of the proposed OTA.

The simulated equivalent input noise of the proposed OTA with $I_{B}$ = 1 nA is shown in Figure 7. The noise spectrum exhibits the typical behavior of CMOS analog circuits, where flicker noise dominates at very low frequencies and gradually decreases with increasing frequency. At approximately 0.1 Hz, the input-referred noise is around 7.3 μV/√Hz, mainly due to the 1/f noise component of the MOS transistors operating in the subthreshold region. As the frequency increases, the noise rapidly decreases and approaches a nearly flat thermal noise floor of about 1.55 μV/√Hz above roughly 10 Hz. This noise level remains relatively constant up to 1 kHz, indicating that thermal noise becomes the dominant component in this region. The obtained noise performance is acceptable for low-frequency biomedical signal acquisition, such as ECG and EEG.

**Figure 8:**
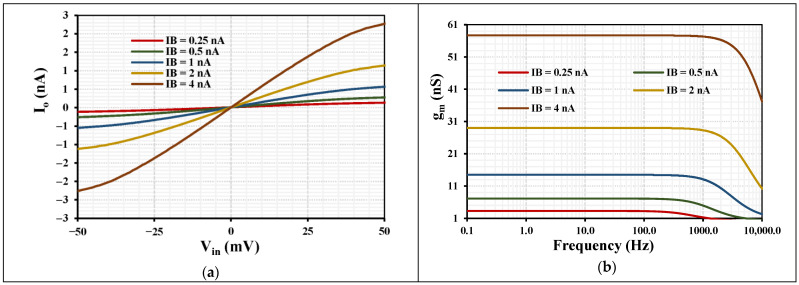
Simulated characteristics of the OTA: (**a**) DC transfer characteristic for a short-circuited output at different bias currents $I_{B}$; (**b**) transconductance $g_{m}$ versus frequency for different bias currents.

Figure 8a illustrates the DC transfer characteristic of the OTA with $I_{B}=0.25,\;0.5,\;1,\;2,$ and 4 nA and with the output short-circuited, showing a nearly linear relationship between input voltage and output current within the input range of ±50 mV. The slope of the characteristic, which corresponds to the transconductance, increases with the bias current $I_{B}$, demonstrating the electronic tunability of the OTA gain. Figure 8b presents the frequency response of the transconductance $g_{m}$ for various bias currents. The OTA maintains a relatively constant $g_{m}$ over a wide low-frequency range, with a slight roll-off at higher frequencies, indicating good stability and bandwidth for low-frequency analog filtering applications. As expected, higher bias currents result in larger transconductance values, which enables dynamic control of filter parameters such as center frequency and bandwidth. The filter, biased with $I_{B}=1\;\mathrm{nA}$, exhibits an ultra-low power consumption of only 4.75 nW.

**Figure 9:**
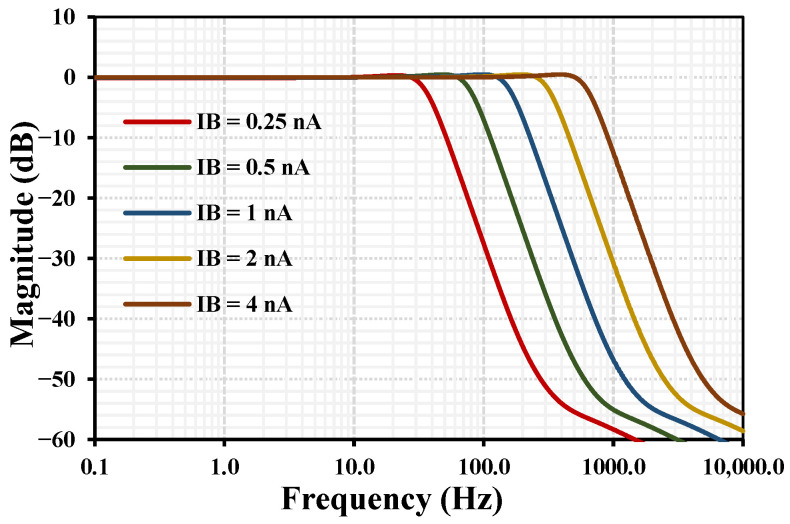
Simulated frequency response of the low-pass filter with various $I_{B}$.

Figure 9 shows the simulated frequency response of the third-order LPF for various bias currents $I_{B}=0.25,\;0.5,\;1,\;2,$ and 4 nA. The −3 dB cutoff frequencies are approximately 37, 82, 167, 335, and 668 Hz, demonstrating a wide tunability range. This tunability effectively covers the frequency spectrum of most biomedical signals, making the filter highly suitable for biosignal processing applications. The impact of the phase error shown in Figure 6 on the third-order Butterworth filter response was analyzed. Due to the simple structure of the proposed filter the phase error in the integrator can lead only to a slight deviation from the ideal maximally flat magnitude response. The overall performance is robust, with only a 0.4 dB gain peaking in the passband. Given the ultra-low-power constraints (1.75 nW per OTA) and the low-frequency nature of biomedical signals, this trade-off is acceptable and does not compromise the functionality of the system.

**Figure 10:**
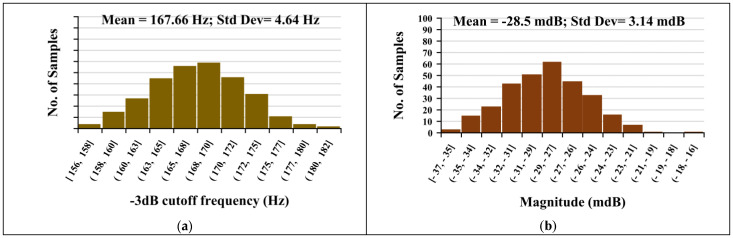
Monte Carlo analysis of LPF: (**a**) −3 dB bandwidth and (**b**) low-frequency gain ($I_{B}=1$ nA, 400 runs).

Monte Carlo mismatch analysis was performed for the filter with $I_{B}=1$ nA. A total of 400 runs were simulated to assess variations in the −3 dB bandwidth and low-frequency gain. The histograms in Figure 10 show that both metrics exhibit only minor deviations, with mean values of 167.66 Hz and −28.5 mdB and standard deviations of 4.64 Hz and 3.14 dB, respectively. These results confirm that the proposed LPF is robust to transistor mismatch and maintains stable performance under typical process variations.

**Figure 11:**
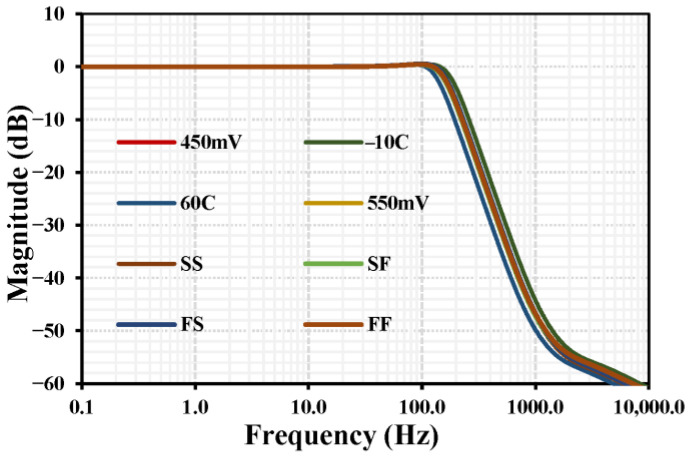
PVT corner analysis of the LPF.

Figure 11 shows the PVT (process, voltage, and temperature) corner analysis of the MOS transistors for the four standard process corners: Fast NMOS/Fast PMOS (FF), Fast NMOS/Slow PMOS (FS), Slow NMOS/Fast PMOS (SF), and Slow NMOS/Slow PMOS (SS). Voltage corners of 450 mV and 550 mV and temperature corners of −10 °C and 60 °C were considered. The curves are closely spaced, indicating that the filter maintains consistent performance across all corners and confirming the robustness of the design.

**Figure 12:**
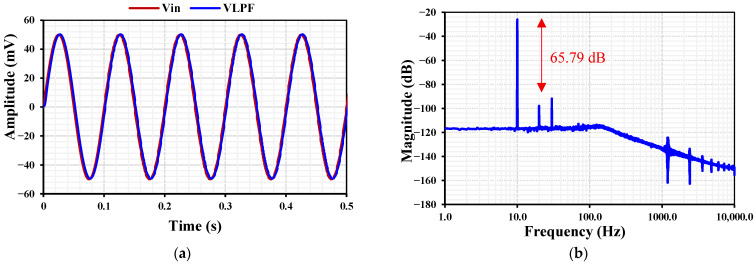
Transient response (**a**) and output spectrum (**b**) of the LPF with $I_{B}=1\;\mathrm{nA}$ for a 10 Hz, 50 mV input signal.

The transient response of the LPF with $I_{B}=1\;\mathrm{nA}\;$ for a 10 Hz, 50 mV amplitude input signal is shown in Figure 12a, while the corresponding output spectrum is presented in Figure 12b. The filter exhibits an exceptionally low total harmonic distortion (THD) of approximately 0.059%, demonstrating excellent linearity for low-frequency biosignal applications.

**Figure 13:**
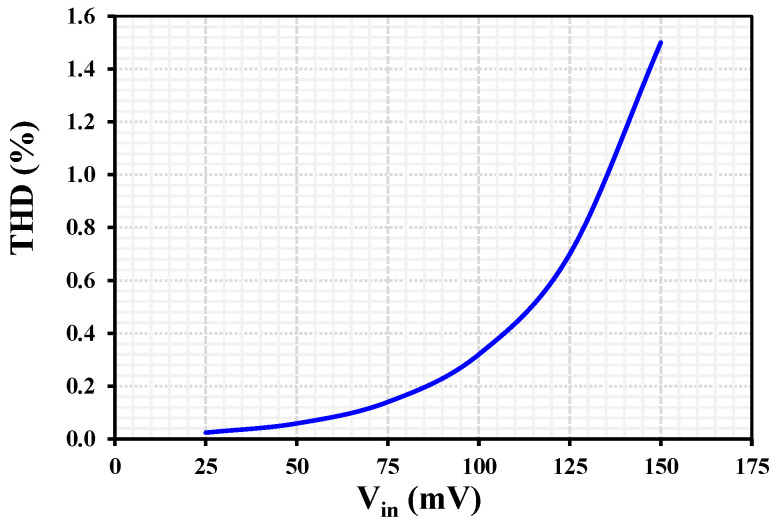
Total harmonic distortion versus input signal amplitude at 10 Hz for $I_{B}=1$ nA.

The THD as a function of the input signal amplitude for I_B = 1 nA at a frequency of 10 Hz is shown in Figure 13. The results indicate that the THD reaches 1% for an input signal amplitude of 137 mV.

**Figure 14:**
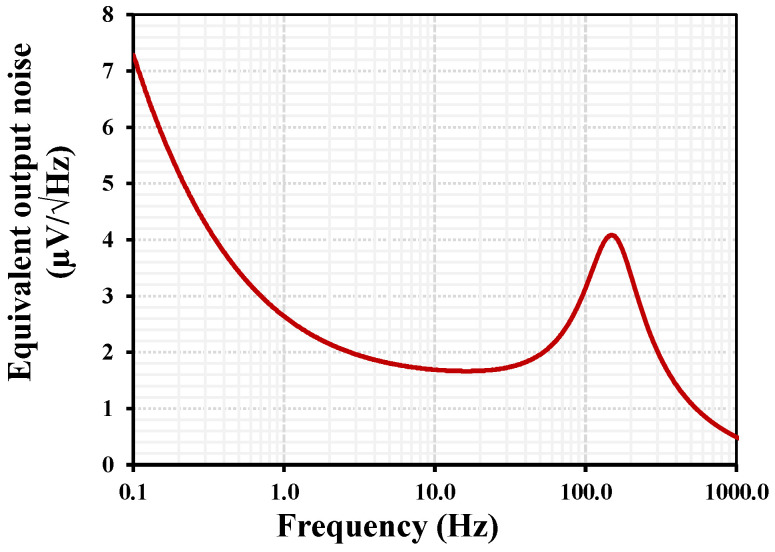
Equivalent output noise of the LPF for $I_{B}=1$ nA.

Figure 14 shows the simulated total equivalent output noise of the LPF for $I_{B}=1$ nA. The output-referred noise integrated over the frequency range from 0.1 to 167 Hz is 38.3 μV. Based on this value, the dynamic range (DR) was calculated to be 65.3 dB for 1% THD. This dynamic range is suitable for biomedical signal acquisition applications.

**Figure 15:**
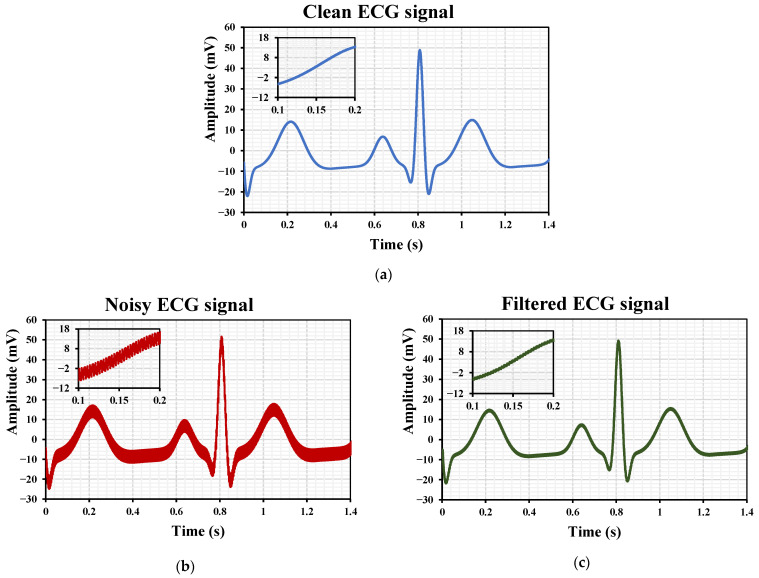
LPF processing of ECG: (**a**) clean signal, (**b**) noisy signal, (**c**) filtered output.

Figure 15 illustrates the use of the third-order LPF for ECG signal processing. The clean ECG signal is shown in Figure 15a. A 3 mV, 300 Hz noise component was added to produce the noisy ECG signal in Figure 15b, which was applied to the LPF input. Figure 15c shows the filtered output, demonstrating effective noise suppression while preserving the ECG waveform. The effectiveness of the noise suppression in Figure 15c is defined by the filter’s ability to attenuate the 300 Hz out-of-band noise component. Given the 167 Hz cutoff frequency and the 60 dB/dec roll-off of the third-order Butterworth topology, the high-frequency interference is significantly suppressed. From the filter characteristics, the signal attenuation for 300 Hz is −17 dB for $I_{B}$ = 1 nA. The preservation of the ECG waveform is quantified by comparing the morphological features of the filtered signal with the original clean signal. Since the critical diagnostic components of the ECG, such as the QRS complex, reside within the filter’s passband, their amplitude and temporal characteristics are maintained with minimal distortion, ensuring the integrity of the clinical information.

The proposed third-order low-pass filter is compared with previously reported low-pass filters, as summarized in Table 1. Recently published works in [24,25,26,27] are selected for comparison. These filters are based on G_m_-C structures. In addition, filters employing OTAs similar to those used in the proposed design, as reported in [28,29], are also included for comparison. Since the filter orders differ, a figure of merit (FOM) is employed to enable a fair comparison [25,26,28]. The proposed low-pass filter achieves the best FOM among the compared works, except for [27]. However, the proposed filter offers the advantages of simple electronic tunability and ease of implementation of the structure. Moreover, the circuit demonstrates superior dynamic range (DR) compared with all referenced works, making it well suited for biomedical signal processing applications.

## 4. Conclusions

In this paper, an ultra-low-power third-order LPF based on a novel bulk-driven OTA architecture was presented. The circuit was designed and verified in a 65 nm CMOS process, achieving a power consumption of only 4.75 nW at a ±0.25 V supply voltage. The most important essence of this research is the demonstration of a high-order, electronically tunable filter that overcomes the voltage headroom challenges of advanced CMOS nodes while maintaining sub-nW power per pole. By leveraging subthreshold operation and strategic biasing, the proposed design offers a 167 Hz bandwidth suitable for ECG monitoring, with effective noise rejection and high linearity. This work proves that advanced 65 nm technology can be effectively utilized for nano-power analog signal processing, providing a critical building block for self-powered implantable and wearable medical devices. While current results are based on high-accuracy simulations, they establish a robust framework for future physical implementation

## Figures and Tables

**Table 1:** Performance comparison of the proposed filter and previous low-pass filters.

Symbol	This Work	[24] 2025	[25] 2025	[26] 2025	[27] 2024	[28] 2020	[29] 2019
VDD [V]	0.5	0.5	1.2	1	0.5	0.5	1.8
Tech [nm]	65	180	180	55	180	180	180
Order (N)	3	4	3	2	4	4	4
Structure	OTA-C	Gm−C	Gm−C	Gm−C	Gm−C	OTA-C	OTA-C
Cutoff frequency [Hz]	167 (Tunable 37–668)	240 (Tunable 29–890)	150	100	100	250 (Tunable 100–250)	50 (Tunable 1–70)
DC gain (dB)	0	−1.4	0.18	−197m	−14.7	−190m	0
Linearity	THD = 1%@137 mV	HD3= −150 dB@100mV	HD3= −151.6 dB@110 mV	HD3= −140 dB@230 mV	THD= −152.5 dB@40 mV	THD = −142.53 dB@50 mV	THD = 1%@98 mV
Input-referred noise [µVrms]	38.3	154.7	43	104	81	39.92	109
DR [dB]	65.3	53.6	65.15	63.88	50.26	62.1	56.06
Power (P) [nW]	4.75	13.3	16.3	4.6	0.224	208	570
FOM [fJ]	5.15	29.4	20	14.71	1.71	163	4480

Note: FOM $=\frac{P}{N\times BW\times DR}.$

## Data Availability

Dataset available on request from the authors.
